# Case Series of Jamestown Canyon Virus Infections with Neurologic Outcomes, Canada, 2011–2016

**DOI:** 10.3201/eid3005.221258

**Published:** 2024-05

**Authors:** Vanessa Meier-Stephenson, Michael A. Drebot, Kristina Dimitrova, Melanie DiQuinzio, Kevin Fonseca, David Forrest, Todd Hatchette, Muhammad Morshed, Glenn Patriquin, Guillaume Poliquin, Lynora Saxinger, Bouchra Serhir, Raymond Tellier, Christian Therrien, Linda Vrbova, Heidi Wood

**Affiliations:** University of Alberta, Edmonton, Alberta, Canada (V. Meier-Stephenson, L. Saxinger);; Public Health Agency of Canada, Winnipeg, Manitoba, Canada (M.A. Drebot, K. Dimitrova, G. Poliquin, L. Vrbova, H. Wood);; Dalhousie University, Halifax, Nova Scotia, Canada (M. DiQuinzio, T. Hatchette, G. Patriquin);; Alberta Precision Labs, Calgary, Alberta, Canada (K. Fonseca);; University of British Columbia, Nanaimo, British Columbia, Canada (D. Forrest);; British Columbia Centre for Disease Control, Vancouver, British Columbia, Canada (M. Morshed);; Institut National de Santé Publique du Québec, Québec City, Quebec, Canada (B. Serhir, C. Therrien);; McGill University Health Centre, Montreal, Quebec, Canada (R. Tellier)

**Keywords:** Jamestown Canyon virus, JCV, arboviruses, California serogroup viruses, arboviral encephalitis, mosquito-borne viruses, meningitis/encephalitis, vector-borne infections, viruses, zoonoses, Canada

## Abstract

Jamestown Canyon virus (JCV) is a mosquitoborne orthobunyavirus in the California serogroup that circulates throughout Canada and the United States. Most JCV exposures result in asymptomatic infection or a mild febrile illness, but JCV can also cause neurologic diseases, such as meningitis and encephalitis. We describe a case series of confirmed JCV-mediated neuroinvasive disease among persons from the provinces of British Columbia, Alberta, Quebec, and Nova Scotia, Canada, during 2011–2016. We highlight the case definitions, epidemiology, unique features and clinical manifestations, disease seasonality, and outcomes for those cases. Two of the patients (from Quebec and Nova Scotia) might have acquired JCV infections during travel to the northeastern region of the United States. This case series collectively demonstrates JCV’s wide distribution and indicates the need for increased awareness of JCV as the underlying cause of meningitis/meningoencephalitis during mosquito season.

Jamestown Canyon virus (JCV) is a mosquitoborne arbovirus belonging to the California serogroup (CSG) viruses (genus *Orthobunyavirus*, family Peribunyaviridae). JCV was first isolated from pooled *Culiseta inornata* mosquitoes in 1961 in Jamestown Canyon, Colorado, USA (*1*). JCV transmission occurs through bites during blood meal acquisition by *Aedes*, *Culesita*, *or Anopheles* mosquitoes, which have wide geographic ranges across North America (*1*–*3*). Despite the existence of JCV in mosquito and mammal hosts, the first human cases of JCV-associated illness were not recognized until 1980 (*4**–**8*).

In North America, the primary amplifying host is thought to be white-tailed deer; however, serologic evidence of JCV has been documented in various domestic and nondomestic animals, including dogs, sheep, mink, cows, horses, foxes, polar bears, elk, and deer (*9*,*10*). In addition, JCV can pass transovarially within the mosquito, which can result in infections early during the mosquito season in May and June; those cases have been documented in different provinces in Canada (*11*; M.A. Drebot, unpub. data).

Human cases of JCV infection are uncommon and have been sporadic. The first JCV infection in Canada was identified in 1981 in an Ontario resident (*8*), and, over the subsequent 4 decades, 1–90 probable and confirmed cases of JCV infection in Canada have been documented each year (*2*,*12*–*15*; M.A. Drebot, unpub. data). In the United States, an average of 16 neuroinvasive JCV cases have been reported each year since 2011 to the Centers for Disease Control and Prevention (CDC) (*16*,*17*). However, JCV infections are likely underdiagnosed and underreported because of asymptomatic or mild manifestations observed in most infected persons (*18*–*22*).

Whereas most exposures to JCV are asymptomatic, clinical manifestations can range from a mild febrile illness to neuroinvasive disease (*23*–*25*). We describe 5 cases of JCV neurologic infections that were reported in the provinces of British Columbia, Alberta, Quebec, and Nova Scotia in Canada during 2011–2016.

## Methods

### Clinical Data and Ethics Statement

We obtained all clinical data through chart review after patient consent within their respective institutions. The Health Canada and Public Health Agency Research Ethics Board provided approval for this research.

### California Serogroup Virus Serology

We screened serum samples for snowshoe hare virus (SSHV) and JCV virus antibodies by using CDC-based or in-house IgM capture ELISAs, as previously described (*26*). We used plaque reduction neutralization tests (PRNTs) to confirm JCV infections and CSG virus exposures (*25*,*27*). We considered the titration endpoint to be the highest dilution of a patient’s serum that inhibited >90% of plaque formation relative to virus controls and a serum titer of >1:20 to be positive. We used endpoint titrations to discriminate cross-reactivity between related CSG viruses.

### Case Definitions

A confirmed case of JCV infection is defined by the Public Health Agency of Canada as clinical illness occurring when and where transmission is likely and laboratory identification of either JCV nucleic acid in blood or cerebrospinal fluid (CSF), a JCV-specific PRNT with >4-fold increase in titer between paired acute and convalescent serum samples (ideally collected >2 weeks apart), or the presence of JCV IgM in a CSF sample and a PRNT titer of >1:20 in a serum sample (Appendix) (*2*). A probable case is defined as clinical illness accompanied by the presence of JCV-specific IgM and a PRNT titer of >1:20 in 1 serum sample. The definitions are similar to those in the CDC guidelines (*28*).

## Cases

### Case 1

On June 23, 2013, a previously healthy 66-year-old man from the Nanaimo region, British Columbia, was admitted to a hospital because of a 2-day history of fever, fatigue, and cough and subsequent vomiting and diarrhea. He was confused at admission and had a Glasgow coma scale score of 11, but his condition deteriorated, requiring transfer to the intensive care unit for worsening encephalopathy and respiratory distress that ultimately required intubation. A computed tomography (CT) scan of his head revealed no abnormalities, and a lumbar puncture was performed; CSF had 50 × 10^9^ leukocytes/L (85% lymphocytes), 3 mmol/L glucose (reference range 2.2–3.9 mmol/L), and 1.45 g/L protein (reference range 0.2–0.45 g/L) (Table). The patient had recurrent myoclonus and possible tonic-clonic seizures; an electroencephalogram showed generalized slowing with bifrontal spike and slow discharges but no clear electrographic seizure activity. Magnetic resonance imaging (MRI) of the brain showed nonspecific white matter hyperintensities and changes consistent with inflammation and was atypical for ischemic injury.

**Table:** CSF and serologic characteristics of patient samples in case series of Jamestown Canyon virus infections with neurologic outcomes, Canada, 2011–2016*

Case no.	Province	Date	Age, y/sex	CSF parameters†	JCV IgM, blood	PRNT titer, acute serum	PRNT titer, convalescent serum	IgM or PRNT titer, CSF
1	BC	Jun 2013	66/M	50 × 10^9^ leukocytes/L, 85% lymphocytes; 1.45 g/L protein; 3.0 mmol/L glucose	+, Jun 23; +, Jun 30	JCV, 1:40, Jun 23; JCV, 1:80, Jun 30; no SSHV reported	NA	JCV, + IgM, 1:10 PRNT, Jun 26; SSHV, – IgM/PRNT
2	AB	Sep 2011	48/M	246 × 10^9^ leukocytes/L, 1% neutrophils, 98% lymphocytes; 1.73 g/L protein; 4.0 mmol/L glucose	+, Sep 27	JCV, 0, Sep 27; SSHV, 0; Sep 27	JCV, >1:80, Oct 4; SSHV, 0; Oct 4	JCV, + IgM, Sep 26
3	AB	Sep 2013	68/M	321 × 10^9^ leukocytes/L, 52% neutrophils, 36% lymphocytes; 0.54 g/L protein; 4.7 mmol/L glucose	+, Sep 26	JCV, 0, Sep 26; SSHV, 0; Sep 26	JCV, 1:40, Nov 5; SSHV, 0; Nov 5	NA
4	QC	Aug 2011	53/M	5 × 10^9^ leukocytes/L, 0.10 g/L protein, 5.3 mmol/L glucose	+, Aug 26; +, Sep 19; +, Sep 30	JCV, 1:640, Aug 26; SSHV, 1:160; Aug 26	JCV, 1:2,560, Sep 19; SSHV, 1:640; Sep 19; JCV, 1:640, Sep 30; SSHV, 1:160, Sept 30	JCV, + IgM, – PRNT, Aug 26; JCV,+ IgM, 1:16 PRNT, Sep 6
5	NS	Jun 2016	70/M	41 × 10^9^ leukocytes/L, 100% lymphocytes; 0.67 g/L protein; 3.2 mmol/L glucose	+, JCV; +, SSHV	JCV, 1:320, Jul 29; SSHV, 1:40; Jul 29	JCV, 1:1,280, Aug 11; SSHV, 1:80, Aug 11	JCV, + IgM, 1:4 PRNT, Jul 27; SSHV, – IgM/PRNT

*AB, Alberta; BC, British Columbia; CSF, cerebrospinal fluid; JCV, Jamestown Canyon virus; NA, not applicable; NS, Nova Scotia; PRNT, plaque reduction neutralization test; QC, Quebec; SSHV, Snowshoe Hare virus; +, positive; –, negative. †Reference ranges for CSF: leukocyte count, 0–5 × 10^9^ cells/L; protein, 0.2–0.45 g/L; glucose, 2.2–3.9 mmol/L.

Serum and CSF samples were negative for cryptococcal antigen, and bacterial and fungal culture results were also negative. In CSF samples, results of PCR testing for herpes simplex virus (HSV) and varicella zoster virus and reverse transcription PCR for enteroviruses were negative. Serum samples were negative for *Borrelia*, *Bartonella*, *Leptospira*, *Mycoplasma, Francisella*, *Coxiella burnetii*, *Toxoplasma*, HIV, West Nile virus (WNV), hepatitis viruses, measles, and parvovirus. PRNTs for JCV IgM had positive titers of 1:40 on June 23 and 1:80 on June 30. Further testing documented that both serum and CSF samples had JCV-specific neutralizing antibodies (Table).

Six months later, the patient reported ongoing issues with coordination and balance. He also noted continuing problems with concentration, short term memory, and depression and was consequently unable to resume work.

### Cases 2

An otherwise healthy 48-year-old man from Alberta, manifested bilateral retroorbital and temporal pain in late September 2011. He also had left-sided arm and leg parasthesias lasting ≈5 minutes. Exposures before onset of symptoms included travel to Cadomin and Wabamun in Alberta (Figure). He also traveled to his vacation home in Las Vegas, Nevada, USA, where his headache became persistently severe the next day, and vomiting, a stumbling gait, and a spinning sensation developed. He sought medical care but was presumed to have influenza and was discharged. He displayed confusion and unusual behavior, such as calling his wife but not speaking, over the next 4 days. He returned to Alberta, where he was found to be dazed and walking oddly with evident confusion and had several episodes of short-term memory loss. His retroorbital headache and paraesthesia of his right thigh were still present. He was taken to an emergency department (ED). After unremarkable results for CT head scan and symptom improvement were observed, he was again discharged. He was unreachable for a period the next day and unable to speak when he answered his phone. He was found in his vehicle on the side of the road, pulled over and vomiting, and he indicated that he had a headache. In the ED, a repeat CT head scan showed a vague 5-mm low-density focus within the right parietal lobe, but no other acute changes were observed. His peripheral complete blood cell count, electrolytes, and renal and liver function measurements were all within reference ranges. CSF revealed 246 × 10^9^ leukocytes/L (98% lymphocytes), 4 mmol/L glucose, and 1.73 g/L protein. Bacterial culture results were negative. CSF was negative for enterovirus/parechovirus, varicella virus, and HSV. He was discharged after 3 days with a plan for a follow-up MRI as an outpatient. While at work the next day, he became uncommunicative and had a headache and right-sided paraesthesias and was hospitalized. His neurologic exam revealed depressed mental status and poor concentration. He had asymmetric reflexes (left side reflexes were greater than the right), spastic tone, and downgoing plantar reflexes. Bloodwork and CSF results were essentially unchanged. He had an electroencephalogram, which showed frontal intermittent rhythmic delta activity; an MRI showed multiple nonspecific hyperintensities in the right frontal cortex and right splenium of the corpus callosum.

**Figure:**
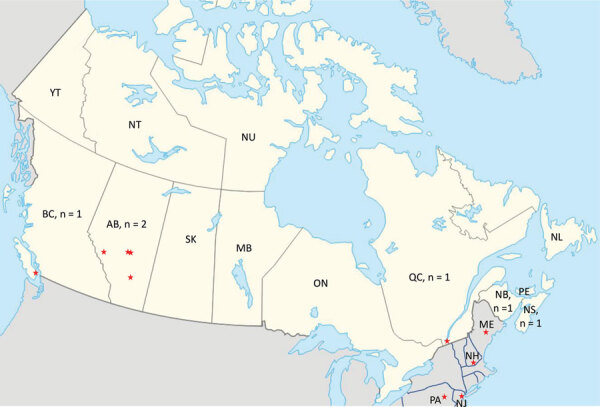
Regions of potential virus exposure in case series of Jamestown Canyon virus infections with neurologic outcomes, Canada, 2011–2016. Red stars indicate regions reported by each symptomatic patient with JCV infection across Canada (yellow area) and northeastern United States (gray area). Numbers indicate the number of case-patients residing in specific provinces of Canada. Some case-patients reported >1 potential exposure sites. Image was adapted from Wikipedia Commons (https://commons.wikimedia.org). AB, Alberta; BC, British Columbia; MB, Manitoba; ME, Maine; NB, New Brunswick; NH, New Hampshire; NJ, New Jersey; NL, Newfoundland and Labrador; NS, Nova Scotia; NT, Northwest Territories; NU, Nunavut; ON, Ontario; PA, Pennsylvania; PE, Prince Edward Island; QC, Quebec; SK, Saskatchewan; YT, Yukon.

Acute and convalescent serum samples were positive for JCV IgM, and a convalescent PRNT had a titer >1:80. In February 2012, a repeat lumbar puncture was performed; CSF was positive for JCV IgM. The patient made a full recovery without any further sequelae.

### Case 3

A 65-year-old man with a history of Merkel cell cancer, parotidectomy, well-controlled type 2 diabetes, and hypertension manifested acute onset of a dull, unilateral frontal headache during mid-September 2013 in Alberta. Two weeks before onset of symptoms, the patient had been golfing and camping in Three Hills, Alberta, and recalled having received several mosquito bites (Figure). Chills and sweats accompanied by several episodes of vomiting developed 2 days after headache onset. He sought care at an ED 4 days later because of a persistent headache and fever of 38.2°C. A preliminary examination, including bloodwork for temporal arteritis, was negative. Later that evening, he became progressively confused with fluctuating arousal. A CT head scan showed no intracranial pathology, and normal sinuses were observed. CSF showed pleocytosis; 320.6 × 10^9^ leukocytes/L (52% neutrophils, 36% lymphocytes) was observed. High levels of polymorphonuclear cells but no organisms were seen after Gram staining. Glucose level was 4.7 mmol/L; protein was 0.54 g/L. Peripheral blood showed a leukocyte count of 14.6 × 10^9^ cells/L (12.4 × 10^9^ neutrophils/L); hemoglobin, platelets, electrolytes, and renal function measurements were within reference ranges. The peripheral glucose level was 12.6 mmol/L, C-reactive protein was 9.0 mg/L, and erythrocyte sedimentation rate was 34 mm/h. Serum samples were negative for HIV, WNV, Lyme disease bacteria, and cytomegalovirus; a nasopharyngeal swab test was negative for respiratory viruses; and CSF was negative for HSV, varicella zoster virus, and enterovirus/parechovirus. All blood and CSF samples had negative bacterial and fungal cultures. Arbovirus serologies (including JCV and SSHV) revealed the presence of JCV IgM in both acute and convalescent serum samples and a 4-fold diagnostic increase in virus-specific neutralizing antibody titers by PRNT (Table**)** (*2*). PRNT results were negative for SSHV. The patient began manifesting postencephalitic fatigue and possible seizure-like episodes; subsequently, normal pressure hydrocephalus developed, requiring a ventriculoperitoneal shunt.

### Case 4

On August 20, 2011, an otherwise healthy 53-year-old man from Montreal, Quebec, sought care because of a 2-day history of fever, fatigue, left-sided neck swelling and pain, and a pruritic rash on his lower back, buttocks, and genitalia. His leukocyte count was slightly elevated at 12.2 x 10^9^ cells/L. His throat was swabbed, and testing later showed a negative result for *Streptococcus*, but he had been given penicillin and antiinflammatory agents at discharge from the ED. He returned to the ED on August 24 because of ongoing fevers, a worsening rash, and 48 hours of increasing shortness of breath, headache, sore throat, and conjunctivitis; he was subsequently admitted. He reported a camping trip in Maine and New Hampshire, USA, during July 31–August 12 but did not recall having any insect bites or contact with ill persons. Bloodwork at admission showed 11.1 × 10^9^ leukocytes/L, 81 × 10^9^ platelets/L, and renal and liver function measurements within reference ranges. Within 24 hours of admission, he became confused, and worsening hypotension and dyspnea developed, requiring intubation and vasopressors. He also had pulmonary edema, pericardial effusion, a new right bundle branch block, and an evolving maculopapular rash on the limbs (including palmoplantar rash) and trunk. A lumbar puncture was performed on August 26, and CSF showed 5 × 10^9^ leukocytes/L (1 × 10^9^ lymphocytes/L), 5.3 mmol/L glucose, and 0.10 g/L protein. Results of blood, CSF, urine, and sputum sample cultures were all negative, including for syphilis. Acute and convalescent serum samples tested negative for *Rickettsia*, *Borrelia*, WNV, Powassan virus, western equine encephalitis virus, and eastern equine encephalitis virus; *Anaplasma phagocytophilum* serology results were positive (indirect immunofluorescence assay titer 1:256), which remained the same throughout the course of illness, suggesting prior exposure.

Serum and CSF samples were positive for JCV IgM by using ELISA; serum samples showed a 4-fold increase (1:640 to 1:2,560) in PRNT titers, then a subsequent decline to 1:640 (Table). Seroconversion of neutralizing antibodies in paired CSF samples was observed by using PRNT, and JCV was distinguished from an SSHV infection by the 4-fold difference in PRNT titer between the 2 viruses (Table).

The patient had confusion and hypoactive delirium throughout his hospitalization. He was discharged on September 21 but continued to have short-term memory loss, expressive aphasia, and some muscle pain. Six months later, his expressive aphasia persisted, but the other symptoms had dissipated.

### Case 5

In mid-June 2016, an otherwise healthy 70-year-old man with psoriasis (no immunotherapies) sought care at an ED in Nova Scotia after a fall; he had a 3–4-day history of a frontal headache, episodic dizziness, and nausea with vomiting. Approximately 2.5 weeks before ED admission, the patient had traveled in the United States for 10 days, visiting New Hampshire, Pennsylvania, and New Jersey. At admission, he was somnolent and febrile (38.6°C) and had a blanching macular rash on his trunk and petechiae on the dorsa of his feet. His speech was slow, and he had impaired concentration, but no nuchal rigidity was present; the remaining neurologic exam was unremarkable. Empiric treatment was initiated with ceftriaxone, ampicillin, vancomycin, and acyclovir. CT and MRI head scans did not demonstrate abnormalities.

Bloodwork showed mild anemia and leukopenia (2.5 × 10^9^ leukocytes/L; nadir 0.5 × 10^9^ leukocytes/L) and thrombocytopenia (73 × 10^9^ platelets/L; nadir 12 × 10^9^ platelets/L); he recovered spontaneously from those conditions within 2 weeks. The patient had hepatic inflammation; alanine aminotransferase level was 74 U/L (peaking at 147 U/L), aspartate aminotransferase was 151 U/L (peaking at 860 U/L), and lactate dehydrogenase was 573 U/L (peaking at >2,500 U/L). Ferritin was elevated at 88,933 μg/L, and creatinine phosphokinase was high at 3,454 U/L.

A lumbar puncture was performed several days after symptom manifestation; CSF had a leukocyte count of 41 × 10^9^ cells/L (100% lymphocytes), glucose level of 3.2 mmol/L, and elevated protein level of 0.67 g/L. CSF and blood cultures were negative. Serologic tests and confirmatory diagnostics for HIV, parvovirus B19, Lyme disease, WNV, and Powassan virus were negative. Acute serum samples were positive for JCV IgM, which was confirmed with a PRNT titer of 1:320; convalescent serum samples showed a 4-fold increase in titer to 1:1,280. CSF was also positive for JCV (PRNT titer of 1:4) (Table).

The patient defervesced by day 2 but reported diffuse myalgias, although his headache was improving. His hospitalization was further complicated by a pulmonary embolism from which he recovered. After 5 weeks of rehabilitation and resolution of his symptoms, the patient was discharged. Upon follow-up, he was found to have made a full recovery.

## Discussion

We describe 5 cases of JCV-associated neurologic disease in patients from British Columbia, Alberta, Quebec, and Nova Scotia in Canada during 2011–2016 and indicate the regions of all potential exposures for each of those cases (Figure). JCV has been shown to circulate across Canada and the United States in various studies (*2*,*7*–*13*,*16*,*20*,*21*,*27*; M.A. Drebot, unpub. data). JCV was first identified as an emerging mosquitoborne pathogen in the early 1980s in both Canada and the United States (*8*). However, of the 23 cases of CSG virus infections identified in Canada during 1978–1989, only 3 were caused by JCV; 18 were caused by SSHV, and 2 had undetermined causes (M.A. Drebot, unpub. data). When CSG virus testing resumed in Canada in 2005, most CSG virus infections were shown to be caused by JCV (*2*), a trend that continues and indicates the emergence of JCV as the primary CSG virus causing infection in Canada (*13*). Although SSHV cases continue to be identified, JCV exposure rates, resulting in both mild and severe illness, appear to have increased. Whether this increase is because of greater JCV circulation, enhanced diagnostic procedures, or other factors warrants further study.

A literature review of encephalitis in Canada highlighted the number of encephalitis cases without a known etiology, suggesting the possibility of a higher prevalence of arbovirus infections than previously thought (*29*). In the appropriate setting, arboviruses, including CSG viruses, should be added to the differential diagnosis of a patient manifesting encephalitis during the mosquito season.

JCV infections in humans can occur throughout the mosquito season and typically display a bimodal pattern. Infections in late spring (May/June) support the concept of transovarial maintenance and the possibility of vertically-infected mosquitoes transmitting virus early during the mosquito season (*30*,*31*). A second peak of infections typically occurs during the late summer and fall months. Case-patients from British Columbia and Nova Scotia had infections that correlated with JCV exposures in late spring (June), whereas the remaining 3 case-patients in this series had exposures during the summer/fall months. Cases of CSG virus infections early during the mosquito season have been documented in Canada; a total of 9 cases of SSHV and JCV infections were identified in the months of May and June during 1978–1989 (*11*; M.A. Drebot, unpub. data). The bimodal peak of arboviral disease likely reflects both transovarial transmission and natural cycling of specific mosquito species that transmit CSG viruses at different times during the mosquito season (*20*,*32*). Because of the various mosquito species responsible for transmitting JCV, exposure can occur throughout the mosquito season (*33*,*34*).

The incubation period for JCV infection is ≈3–14 days. Therefore, the case-patients from Quebec (case 4) and Nova Scotia (case 5) might have been exposed during travel in the northeastern United States. Although no JCV cases had been documented in Maine or New Hampshire at the time of the 2011 case (case 4), subsequent cases were identified in New Hampshire in 2013 and Maine in 2017 (*16*). According to his date of return to Montreal, symptom onset, and the JCV incubation period, case-patient 4 might have been exposed at his residence or during his camping trip in the United States. Case-patient 5 had possible exposures in multiple locations in quick succession in 2016 before seeking care at a Nova Scotia hospital, including wooded areas of New Hampshire, Pennsylvania, and New Jersey, USA; he could have conceivably been infected in any of those US states or in his home province. One case of neuroinvasive JCV infection occurred in New Hampshire in 2013; although no autochthonous neuroinvasive cases of JCV had been previously identified in Nova Scotia, a high (≈21% ) JCV seroprevalence existed in the province (*27*). 

Unlike some of the other CSG serogroup viruses, such as SSHV and La Crosse virus (LACV), most symptomatic JCV infections have been identified in adults (*2*,*4*,*20*,*35*). Clinically, JCV infections can be asymptomatic, self-limited febrile illnesses, or cause meningitis/encephalitis syndromes (*1*,*4*,*24*,*25*,*35*–*37*). An upper respiratory prodrome has occasionally been reported (*4*,*32*,*35*). During neuroinvasive disease, CSF typically shows lymphocyte predominance and variable protein and glucose levels; however, our small case series showed considerable variation in CSF profiles (Table).

As indicated in the case definitions, the time and place for virus transmission alludes to evidence that JCV-specific IgM might persist for several months or even years in the serum from patients exposed to CSG viruses (*15*; M.A. Drebot, unpub. data). Persistence of virus-specific IgM in serum samples has been noted for other arboviruses, such as WNV (*38*). As a result, lingering IgM might confound identification of current CSG virus infections when positive serology is documented by using only acute phase serum samples. We observed the presence of JCV IgM in CSF from case-patient 2 several months after symptom onset, a finding previously documented for some WNV patients (*39*).

A diagnostic 4-fold rise in titers for paired acute and convalescent serum samples is typically informative for confirming new or repeat exposures, particularly given the possibility of persistent IgM. CSF from case-patients 1, 2, 4, and 5 had positive JCV IgM or PRNT titers; case-patients 2–5 had a clear 4-fold increase in JSV PRNT titers in convalescent serum samples. Case-patient 1 of the series had repeat serologic tests 1week after collecting the acute sample, which showed an increased titer but was not considered a convalescent sample (which would ideally be taken at 2–4 weeks, but according to the CDC definition, it should be minimum of 8 days later).

For case-patient 4 (Quebec), JCV infection was confirmed through the positive IgM ELISA results, noted seroconversion and diagnostic increases in JCV-specific antibodies obtained by PRNT in both paired serum and CSF, and increase in serum antibodies, even with concomitant *A*. *phagocytophilum* and SSHV seropositivity. Cross reactions in PRNT and particularly ELISA can occur between SSHV and JCV; however, the IgM increase in serum samples, seroconversion in CSF, and >4-fold differences between JCV and SSHV PRNT titers in acute and convalescent serum samples provide strong evidence of JCV exposure. It is possible that *A*. *phagocytophilum* seropositivity reflects either a previous infection or co-infection, because anaplasmosis is a known emerging infection in many regions within Canada, including Quebec (*40*–*42*).

A limitation of this work is the lack of testing for LACV, a CSG virus that has diagnostic similarities and cross-reactivity to other CSG members (*35*). Testing for LACV was not part of the initial arbovirus testing panels because previous serologic screening studies for this virus had been negative among collections of CSG virus–positive serum samples (M.A. Drebot, unpub. data), despite the geographic range of *Aedes triseriatus* mosquitoes, the primary LACV vector, in southern Canada. Future inclusion of LACV testing in the diagnostic algorithms for suspected CSG virus exposures in Canada is warranted given possible expansion and increased prevalence of the virus.

In conclusion, we describe 5 cases of JCV infection that occurred in Canada early during the mosquito season, highlighting the potential for acquisition of this virus throughout the entire mosquito season. The JCV case-patients from British Columbia and Alberta provide further evidence of JCV exposure risk across Canada. The case-patients from Nova Scotia and Quebec, who had a travel history, indicate that JCV needs to be recognized as a possible cause of neuroinvasive disease for travelers in the United States as well as in Canada. 

Appendix Additional information for case series of Jamestown Canyon virus infections with neurologic outcomes, Canada, 2011–2016.
