# Correction: Vol. 30, No. 5

**DOI:** 10.3201/eid3009.C23009

**Published:** 2024-09

**Keywords:** errata, erratum, correction

The name of author Glenn Patriquin was misspelled in Case Series of Jamestown Canyon Virus Infections with Neurologic Outcomes, Canada, 2011–2016 (V. Meier-Stephenson et al.). The article has been corrected online (https://wwwnc.cdc.gov/eid/article/30/5/22-1258_article).

