# Supplementary material for: Case Series of Jamestown Canyon Virus Infections with Neurologic Outcomes, Canada, 2011–2016
Source: Emerg Infect Dis. 2024 May;30(5):874–81. doi: 10.3201/eid3005.221258 (PMC11060468; doi:10.3201/eid3005.221258)
Supplement: Appendix — Additional information for case series of Jamestown Canyon virus infections with neurologic outcomes, Canada, 2011–2016. [file 22-1258-Techapp-s1.pdf]

Article DOI: https://doi.org/10.3201/eid3005.221258
EID cannot ensure accessibility for supplementary materials supplied by authors. Readers who have difficulty accessing supplementary content should contact the authors for assistance.

Case Series of Jamestown Canyon Virus Infections with Neurologic Outcomes, Canada, 2011–2016

Appendix

Public Health Agency of Canada Definition of Jamestown Canyon Virus Infection

Laboratory-confirmed case (recent infection)

Clinical illness[1] occurring when and where transmission is likely[2] with laboratory confirmation of infection by detection of Jamestown Canyon virus (JCV) or Snowshoe hare virus (SSHV) nucleic acid (e.g., by PCR) in an appropriate clinical specimen (e.g., CSF)[3], ≥4-fold rise in virus-specific plaque reduction neutralization test (PRNT) antibody titers detected in paired serum samples (acute- and convalescent-phase serum ideally collected ≥2 weeks apart)[4,5], or virus-specific IgM in a CSF sample[6] and a PRNT titer ≥1:20 in serum.[5]

Probable Case (timing of acquisition cannot be determined)

Clinical illness[1] occurring when and where transmission is likely[2] and the presence of virus-specific IgM and virus-specific PRNT titers ≥1:20 in a single serum specimen.[4,5]

Footnotes

[1]Clinical illness is characterized by a febrile illness of variable severity, which can include neuroinvasive diseases, such as meningitis and encephalitis. Signs and symptoms can include fever, chills, headaches, myalgia, arthralgia, fatigue, nausea, diarrhea, vomiting, abdominal pain, rash, stiff neck, confusion or altered sensorium, visual disorders, altered reflexes, abnormal movements, seizures, and coma.

[2]JCV/SSHV exposure occurs during late spring to early fall in Canada and in the Midwest region of the United States, and year-round in southeastern parts of the United States. If there is a history of travel outside of Canada, consideration should be given to cross-reactivity

between JCV, SSHV, and other California serogroup (CSG) orthobunyaviruses, such as La Crosse virus (LACV). Although LACV has not yet been detected in Canada, the virus and its vector might expand northward because of climate change, and the vector has been detected in Ontario, Quebec, and New Brunswick. LACV occurs in the Midwest and mid-Atlantic regions of the United States.

[3]Reverse transcription PCR of serum or CSF samples is not a sensitive test for CSG viruses because of limited viremia and is only performed under special circumstances. The National Microbiology Laboratory will determine if molecular testing is feasible after serologic testing is complete.

[4]Because of the possibility of persistent IgM in serum samples, the demonstration of a seroconversion is necessary to associate a positive serologic test with a current illness. A second laboratory result with a stable (static or ≤2-fold increase) antibody titer is still suggestive of recent infection but might depend on when the specimen was taken in relation to onset of symptoms (e.g., the rise in titer might be missed because of timing of sample collection). Seroconversion might also be demonstrated by detecting IgM seroconversion in paired serum samples when combined with virus-specific PRNT antibody titers ≥1:20 detected in 1 serum sample.

[5]Because of cross-reactivity between related orthobunyaviruses, either a negative PRNT for a related orthobunyaviruses or a ≥4-fold difference between PRNT antibody titers for the virus of interest and related orthobunyaviruses is required to indicate virus-specific antibodies. Without those results, the case is classified as a CSG virus infection.

[6]Further investigation is required to rule out cross-reactions (e.g., negative results for other IgM antibodies in CSF for arboviruses endemic to the region where exposure occurred are required, or additional serum samples are needed to rule out static titers).
